# hCLE/C14orf166 Associates with DDX1-HSPC117-FAM98B in a Novel Transcription-Dependent Shuttling RNA-Transporting Complex

**DOI:** 10.1371/journal.pone.0090957

**Published:** 2014-03-07

**Authors:** Alicia Pérez-González, Alejandra Pazo, Rosana Navajas, Sergio Ciordia, Ariel Rodriguez-Frandsen, Amelia Nieto

**Affiliations:** 1 Centro Nacional de Biotecnología. C.S.I.C. Darwin 3, Cantoblanco, Madrid, Spain; 2 Ciber de Enfermedades Respiratorias, Madrid, Spain; Korea University, Republic of Korea

## Abstract

hCLE/C14orf166 is a nuclear and cytoplasmic protein that interacts with the RNAP II, modulates nuclear RNA metabolism and is present in cytoplasmic RNA granules involved in localized translation. Here we have studied whether hCLE shares common interactors in the nucleus and the cytosol, which could shed light on its participation in the sequential phases of RNA metabolism. Nuclear and cytoplasmic purified hCLE-associated factors were identified and proteins involved in mRNA metabolism, motor-related proteins, cytoskeletal and translation-related factors were found. Purified hCLE complexes also contain RNAs and as expected some hCLE-interacting proteins (DDX1, HSPC117, FAM98B) were found both in the nucleus and the cytoplasm. Moreover, endogenous hCLE fractionates in protein complexes together with DDX1, HSPC117 and FAM98B and silencing of hCLE down-regulates their nuclear and cytosolic accumulation levels. Using a photoactivatable hCLE-GFP protein, nuclear import and export of hCLE was observed indicating that hCLE is a shuttling protein. Interestingly, hCLE nuclear import required active transcription, as did the import of DDX1, HSPC117 and FAM98B proteins. The data indicate that hCLE probably as a complex with DDX1, HSPC117 and FAM98B shuttles between the nucleus and the cytoplasm transporting RNAs suggesting that this complex has a prominent role on nuclear and cytoplasmic RNA fate.

## Introduction

The hCLE/C14orf166 protein has been shown to be a constituent of nuclear and cytoplasmic protein complexes involved in different phases of the RNA metabolism. We identified the human hCLE protein by its interaction with influenza virus PA polymerase subunit [1] and was later shown to be important for viral polymerase activity and viral particle production [2]. hCLE is a 24 kDa protein that shows a nuclear and cytoplasmic localization [1], it associates with the RNAP II and is present in nuclear sites of RNAP II-directed RNA transcription [3]. Accordingly, hCLE silencing inhibits cellular mRNA transcription by 50%, which causes the down-regulation of an important number of genes [3]. Several proteomic analyses have described hCLE as an important component of nuclear complexes involved in transcriptional-related functions such as the human spliceosome [4], the 7SK snRNA methylphosphate capping complex [5], and the tRNA-splicing ligase complex [6]. They also have shown its association with RNA-maturation related proteins [7]. This information indicates that hCLE is a positive modulator of RNAP II transcription that could be involved in transcription initiation and elongation as well as RNA processing. On the other hand, hCLE has been found in cytoplasmic mRNA-containing kinesin-associated granules present in dendrites, which play a role in local protein synthesis [8]. hCLE has also been described as a component of cytosolic RNA granules that contain ribosomes and that transport specific mRNAs from the cell body to the dendrites in developing brain, allowing for local protein translation at sites distant from the nucleus [9]. In addition, hCLE my have a more general role in viral infections since besides its role on influenza virus replication it has been found associated with nuclear HIV-1 RNA [10], with the mature Hepatitis C virus core protein [11] and recently with the IBV Coronavirus nucleocapsid protein [12].

Since hCLE is distributed in the nucleus and the cytoplasm playing a role in different steps of nuclear and cytoplasmic RNA metabolism such as transcription, maturation and translation, we were interested in the characterization of the hCLE nuclear and cytosolic interacting proteins. This information would provide further evidence about the role of hCLE and would enable the study of whether hCLE shares common interactors in the nucleus and the cytosol, which could shed light on its participation in the sequential phases of RNA metabolism. The results obtained indicate that a complex containing DDX1-HSPC117-FAM98B-hCLE proteins is present in both cellular compartments and moreover their nucleo-cytoplasmic transport requires active RNA transcription, suggesting that this complex has a prominent role on nuclear and cytoplasmic RNA fate.

## Materials and Methods

### Biological Materials

The human HEK293T cell line was provided by J.C. de la Torre and cultivated as described previously [13]. Plasmid pC-TAP, was previously described [14]. The cDNA of hCLE was cloned upstream of the TAP sequence in the pC-TAP plasmid to generate pChCLE-TAP and upstream of the photoactivatable GFP sequence in pC1-PAGFP to generate phCLE-PAGFP. Plasmid pC1-PAGFP was a kind gift from Jennifer Lippincott-Schwartz (Eunice Kennedy Shriver National Institute of Child Health and Human Development, Bethesda, MD), and has been previously described [15]. Actinomycin D was obtained from SIGMA.

### Transfection

Transfection of subconfluent HEK293T cells was carried out using the calcium phosphate method [16]. Four or 30 µg of DNA were transfected per 35 mm or 150 mm culture dishes, respectively. Cells were processed for TAP-tag purification 24 h after transfection.

### Western Blotting

Western blotting was carried out as described previously [17]. The following primary antibodies were used: for hCLE, a rabbit polyclonal antibody (1∶1000) from Abcam; for RNAP II, a monoclonal antibody 8WG16 (1∶500) from Covance; for β-tubulin, a monoclonal antibody (1∶15000) from SIGMA, for DDX-1, a goat polyclonal antibody sc-49817 (1∶200) from Santa Cruz Biotechnology; for HSPC117/C22orf28, a goat polyclonal antibody LS-C139785 (1/3000) from LifeSpan BioSciences; for FAM98B, a rabbit polyclonal antibody HPA008320 (1/500) from Sigma; for α-actin, a rabbit polyclonal antibody (1/200) from Abcam and for S6, a mouse monoclonal antibody 54D2 (1/1000) from Cell Signaling that recognizes total S6 protein.

### Immunofluorescence

Cells were fixed with 3.7% formalin in PBS during 20 min at room temperature and permeabilised for 5 min with 0.5% Triton X-100 in PBS. The preparations were blocked with 1% bovine serum albumin (BSA) in PBS for 30 min and then incubated for 45 min with the following primary antibodies: a rabbit polyclonal antibody against hCLE (1∶1000) [1], a goat polyclonal antibody against DDX1 (1/50), and a goat polyclonal antibody against HSPC117 (1/500). After washing with PBS, the preparations were incubated with the corresponding secondary antibodies and DAPI using the same conditions. Finally, the preparations were mounted in ProLong Gold and incubated for 16 h at room temperature. The analysis of the preparations was done using a BioRad Radiance 2100 laser scanning system on a Zeiss Axiovert 200 microscope. For drug effects on hCLE distribution, HEK293T cells were treated with Actinomycin D at 5 µg/ml in DMEM-10% FBS during 30 min at 37°C. After drug treatment the cells were processed for immunofluorescence as described above.

### Lentiviral Particle Production and Cell Transduction

Lentiviral particles were produced in HEK-293T cells by cotransfection of plasmids encoding short hairpin RNAs (shRNAs) targeting the indicated gene and the plasmids necessary for vesicular stomatitis virus glycoprotein-pseudotyped lentivirus production (pMD2.G, Addgene #12259 and psPAX2, Addgene #12260). Cell supernatants were collected at 40 h posttransfection, filtered through 0.45-µm filters, aliquoted, and kept at −80°C till needed. Lentiviral vectors encoding short hairpin RNAs (shRNAs) targeting the positions 121 to 143 (siCLE.1) and 417–435 (si.CLE.2) in the cDNA of hCLE were generated by insertion of the adaptor primers pair (5′CCGGTAAGATTGAAGACAGAGGGAATTTTTCAAGAGAAAATTCCCTCTGTCTTCAATCTTTTTTTG3′ and 5′AATTCAAAAAAAGATTGAAGACAGAGGGAATTTTCTCTTGAAAAATTCCCTCTGTCTTCAATCTTA3′) and (5′CCGGTCCTGCTTCAGATTCAGCGTTTCAAGAGAACGCTGAATCTGAAGCAGGTTTTT3′ and 5′ AATTCAAAAACCTGCTTCAGATTCAGCGTTCTCTTGAAACGCTGAATCTGAAGCAGG3′ respectively, immediately after the U6 promotor in the AgeI/EcoRI-digested pLKO-puro vector (Addgene # 8453). Control shRNA plasmid expressing a sequence from Thermotoga Maritima was generated using a similar strategy by insertion of the adaptor primer pair (5′CCGGTAATTCTCCGAACGTGTCACGTTTCAAGAGAACGTGACACGTTCGGAGAATTTTTTTG 3′ and 5′AATTCAAAAAAATTCTCCGAACGTGTCACGTTCTCTTGAAACGTGACACGTTCGGAGAATTA 3′). The pCDNA-hCLE silencing resistant mutant was generated by introducing 3 silent mutations at positions 126, 135 and 141 in the target sequence of hCLE cDNA by using the QuickChange™ Site-Directed mutagenesis kit (Stratagene).

### Purification of hCLE-TAP Tagged Protein

hCLE-TAP protein was purified from nuclear or cytosolic cell extracts. In brief, transfected cells were collected in PBS and pelleted by centrifugation, resuspended in a solution containing 10 mM Tris-HCl pH 8.0, 10 mM KCl, 0.1% NP-40, 1 mM EDTA, 1 mM DTT and protease inhibitors (Complete, Roche) and incubated in ice for 5 min. Then, they were centrifuged for 5 min at 3000 rpm at 4°C. The supernatant was recovered and supplemented with NaCl to a final concentration of 150 mM (cytosolic extract). The sedimented nuclei were extracted in a solution containing 20 mM Tris-HCl pH 8.0, 0.4 M NaCl, 10% glycerol, 1.5 mM MgCl_2_, 1 mM EDTA, 1 mM DTT and protease inhibitors (Complete, from Roche) for 30 min in ice with occasional vortexing and centrifuged for 15 min at 15000 rpm at 4°. The supernatant (nuclear extracts) was adjusted to final concentration of NaCl 150 mM, Tris-HCl 10 mM pH 8.0 and NP-40 0.1%. Nuclear or cytosolic extracts were then used for TAP purification as previously described [18].

### Identification of hCLE-associated Proteins

#### In-gel protein digestion and sample preparation for MALDI-TOF/TOF analysis

Bands of interest from silver-stained gels were excised manually and processed automatically in a Proteineer DP (Bruker Daltonics, Bremen, Germany). The digestion protocol used was as reported [19].

#### Protein identification by MALDI-TOF/TOF analysis

For MALDI-TOF/TOF analysis, samples were automatically acquired in an ABi 4800 MALDI TOF/TOF mass spectrometer (Applied Biosystems, Framingham, MA, USA) in positive ion reflector mode (the ion acceleration voltage was 25 kV to MS acquisition and 1 kV to MSMS) and the obtained spectra were stored into the ABi 4000 Series Explorer Spot Set Manager. PMF and MSMS fragment ion spectra were smoothed and corrected to zero baseline using routines embedded in ABi 4000 Series Explorer Software v3.6. Each PMF spectrum was internally calibrated with the mass signals of trypsin autolysis ions to reach a typical mass measurement accuracy of <25 ppm. Known trypsin and keratin mass signals, as well as potential sodium and potassium adducts (+21 Da and +39 Da) were removed from the peak list. To submit the combined PMF and MS/MS data to MASCOT software v.2.2.04 (Matrix Science, London, UK), GPS Explorer v4.9 was used, searching in the non-redundant NCBI protein database (NCBInr 20080421, 6122577 sequences; 2096230148 residues). The following search parameters were used: enzyme, trypsin; allowed missed cleavages, 1; carbamidomethyl cystein as fixed modification by the treatment with iodoacetamide; variable modifications, oxidation of methionine; mass tolerance for precursors was set to ±100 ppm and for MS/MS fragment ions to ±0.8 Da. The confidence interval for protein identification was set to ≥95% (p<0.05) and only peptides with an individual ion score above the identity threshold were considered correctly identified.

#### Sample preparation for LC-MS/MS (liquid chromatography coupled to tandem mass spectrometry) analysis

Nuclear and cytosolic affinity purified samples from hCLE-TAP and TAP (Control) expressing cells, were subjected to methanol-chloroform precipitation to isolate proteins and remove interfering substances. The protein extracts were dissolved in 20 µl of 50 mM TEAB/50% TFE, reduced by 10 mM TCEP, and alkylated by addition of cysteine-blocking reagent MMTS. Samples were further diluted up to 200 µl with 50 mM TEAB and digested with trypsin at an enzyme-to protein ratio of 20∶1, at 37°C overnight. For the purification of the tryptic peptide mixtures, samples were dried and redissolved in 40 µl of 0.2% trifluoroacetic acid in water, and high-capacity OMIX C18 tips were used.

#### Protein identification by LC–MS/MS analysis

The peptide samples were analyzed on a nano liquid chromatography system (Eksigent Technologies nanoLC Ultra 1D plus, AB SCIEX, Foster City, CA) coupled to 5600 Triple TOF mass spectrometer (AB SCIEX, Foster City, CA) with a nanoelectrospray ion source. The analytical column used was a silica-based reversed phase column Chrom XP C18 75 µm×15 cm, 3 µm particle size and 120 Å pore size (Eksigent, AB SCIEX). The trap column was a Chrom XP C18 350 µm×0.5 mm (Eksigent, AB SCIEX), 3 µm particle diameter, 120 Å pore size, switched on-line with the analytical column after a 1 min loading time. The loading pump delivered a solution of 0.1% formic acid in water at 2 µl/min. The nano pump provided a flow-rate of 300 nl/min and was operated under gradient elution conditions, using 0.1% formic acid in water as mobile phase A, and 0.1% formic acid in acetonitrile as mobile phase B, from 2 to 40% B in 100 minutes. Gradient elution was performed according to the following scheme: isocratic conditions of 98% A: 2% B for 1 minute, a linear increase to 30% B in 54 minutes, a linear increase to 40% B in 10 minutes, a linear increase to 90% B in 5 minutes, isocratic conditions of 90% B for 10 minutes and return to initial conditions in 8 min. Injection volume was 5 µl. The mass spectrometer was operated in data-dependent acquisition mode. For TOF scans, the accumulation time was set to 0.250008 s, and the mass range to 350–1250 Da. Peptides with 2–5 charges and signals above 100 cps were selected for fragmentation. Per cycle, up to 15 precursor ions were monitored and excluded for 20 s after one occurrence. The product ion scan was performed with an accumulation time of 0.250008 s, and a mass range of 100–2000 Da in a high sensitivity mode. The total cycle time was 4.05 s. To prevent cross-contamination, two reagent blank samples were injected between samples.

MS and MS/MS data obtained for each sample were processed using Analyst TF 1.5.1 Software (AB SCIEX, Foster City, CA). Raw data were translated to mascot general file (mgf) format and searched against the UniProtKB/Swiss-Prot human database (release 2012_10, October 31) that contains 36962 proteins and their corresponding reversed sequences, using an in-house Mascot Server v. 2.4 (Matrix Science, London, U.K.). Search parameters were set as follows: methylthiocysteine as fixed modification and oxidized methionines as variable one. Peptide mass tolerance was set to 50 ppm and 0.5 Da, in MS and MS/MS mode, respectively and 1 missed cleavage was allowed. Typically, accuracy below 10 ppm was found both for MS and MS/MS spectra. False Discovery Rates (FDR<1%) for peptide identification were manually calculated as follows: after database searching, peptide matches were ordered according to their Mascot scores. This list contained peptide sequences matching either forward or reversed database sequences. Then, a sublist containing less than 1% of peptides matching the reversed sequences was extracted and used for further analysis.

### Gel Filtration

Nuclear and cytoplasmic fractions of HEK293T cells were prepared as described above and applied to a Sephacryl S400 resin equilibrated in the corresponding nuclear or cytosol extraction buffers without DTT and glycerol and calibrated with influenza virus RNPs (3–6 mDa) purified as described [20], ferritin (480 kDa) and catalase (220 kDa), at a sample to bed volume ratio of 1∶100. The void volume was calibrated with dextran blue (MW 2,000,000) that was excluded in the first 6 fractions. The hCLE complexes were analyzed by Western blot.

### RNA Analysis

The RNA associated to hCLE-TAP was obtained from the purified complexes by proteinase K (0.2 mg/ml) and 0.5% SDS treatment in TNE buffer for 1 h at 37°C. After phenol extraction, DNAse (TURBO DNase, Ambion) treatment was performed and the RNAs were precipitated with 3 volumes of ethanol and 20 µg of glycogen. RNAs were labeled by incubation with [γ-^32^P]ATP (250 µCi/ml) and polynucleotide kinase for 1 h at 37°C. After separation from remaining free nucleotide by Illustra MicroSpin G-25 columns (GE-Healthcare), RNAs were analyzed by denaturing polyacrylamide-urea gel electrophoresis and radioactivity was detected in a phosphorimager.

### Live Cell Imaging

HEK293T cells were transfected with the phCLE-PAGFP plasmid and 24 hours post-transfection, used for live cell microscopy using a confocal Leica TCS SP5 unit. This photoactivatable GFP has a particularly dim fluorescence if not photoactivated, but when excited at a wavelength of 405 nm, gives a high peak of emission at 488 nm. A 50 mW blue diode 405 nm laser at 80% transmission was used for photoactivation in selected regions of interest (2 µm diameter) using the photobleaching function of the LAS AF software in time-lapse mode. The 488 nm line of the Argon laser was used at very low transmission percentage (4%) for time-lapse imaging of PAGFP. Photobleaching was not observed during the time-course.

The videos were analyzed with Leica Microsystems LAS AF software (version 2.3.0).

## Results

### HCLE Forms Dimers that are Resistant to Denaturing Conditions

**Figure 1:**
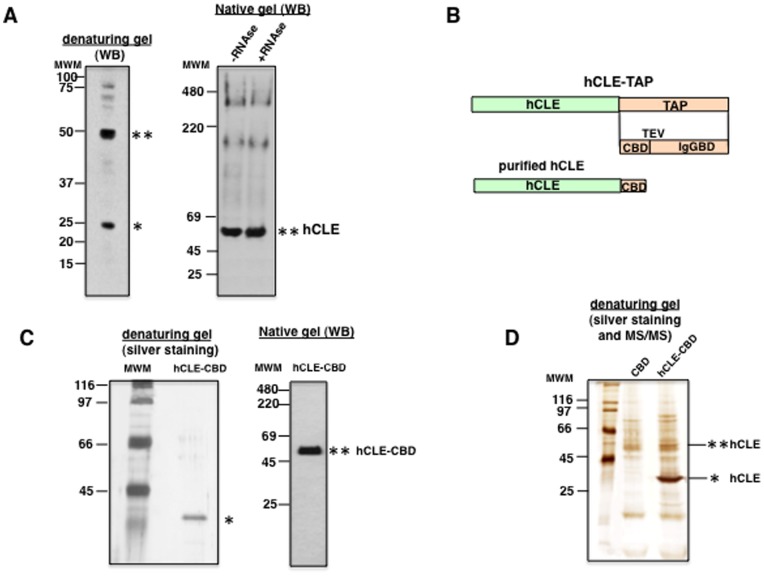
hCLE forms dimers that are resistant to denaturing conditions. (A); Western blot against hCLE of total extracts of HEK293T cells using denaturing (left) or native conditions (right). (B); Diagram showing the scheme of the fusion protein hCLE-TAP with its tags for affinity purification. (C); left, HEK293T cells were transfected with the hCLE-TAP expressing plasmid and total cell extract was used for affinity purification, a sample of the purified protein was analyzed by silver staining in SDS-PAGE gels. Right; The hCLE-CBC purified protein was dialyzed and analyzed in non-denaturing gels followed by Western blot detection. (D). The hCLE-CBD purified protein was subjected to silver staining and bands corresponding to different sizes were excised and analyzed by MS-MS technique. The lines denote hCLE identification by MS-MS technique. (*), denotes hCLE monomer and (**) hCLE dimer.

Earlier reports using a yeast two-hybrid system indicated that the C-terminal part of hCLE (aa 151–244) is able to interact with itself [21]. Previous experiments employing cellular extracts to detect hCLE by Western blotting showed an anomalous migration of hCLE in SDS-denaturing gels according to its predicted molecular weight of 24 kDa. In these assays two main bands detected by the anti-hCLE antibody corresponding to around 24 and 48 kDa were observed (Fig. 1A left panel). These bands could correspond to hCLE monomer and either to an unspecific reaction of the anti-hCLE antibody or to denaturing conditions-resistant dimers of the protein. Thus, we first analyzed the size of the protein in non-denaturing gels treated or untreated with RNAse (Fig. 1A right panel). The smallest anti-hCLE reactive band has a 48 kDa molecular weight, which could correspond to hCLE dimer, also larger anti-hCLE reactive bands were observed in the cell extract, while almost undetectable levels of the hCLE in the monomeric form were observed and identical results were found in the RNAse treated cells. These results indicate that hCLE is present in protein complexes and that the minimal molecular weight of hCLE complexes corresponds to possible hCLE-hCLE dimers. Since the 48 kDa reactive band could be either hCLE dimers or hCLE associated to a protein of a similar molecular weight, we expressed and purified hCLE with a tag that allows its purification. The construct expresses a carboxy-terminal hCLE-TAP (tandem-affinity purification) tagged protein (pChCLE-TAP) and was generated by cloning the cDNA for hCLE upstream of the TAP tag sequence in the pC-TAP plasmid [14]. The TAP tag contains an IgG-binding domain from protein A (IgGBD) and a calmodulin-binding domain (CBD) separated by a TEV protease cleavage site (see a scheme on Fig. 1B). This allows purification by successive IgG-Sepharose and Calmodulin-Agarose affinity chromatography steps. HEK293T cells were transfected with pChCLE-TAP plasmid and total cell extracts were separated and used for TAP purification. The resulting hCLE-CBD purified protein was analyzed by silver staining in SDS-denaturing gels and a main band of around 27 kDa that correspond to the hCLE-CBD protein was observed (Fig. 1C, left). Next the purified hCLE-CBD protein was dialyzed and analyzed in non-denaturing gels followed by Western blot detection (Fig. 1C, right). A band of around 48 kDa that correspond to hCLE dimer was observed indicating that hCLE is capable to form hCLE-hCLE dimers. Finally we further analyzed whether hCLE-hCLE dimers were present in SDS-denaturing gels. To that aim the hCLE-CBD purified protein was subjected to silver staining and bands corresponding to different sizes were excised and analyzed by MALDI-TOF/TOF mass spectrometry (Fig. 1D). Both hCLE bands of 27 and 48 kDa were observed (Table 1), indicating that hCLE-hCLE is capable to form dimers that are resistant to denaturing conditions. Other examples of proteins forming denaturing resistant dimers have been reported and ascribed to unusual structure features including interwoven subunits [22], [23], [24]. Collectively the data indicate that in physiological conditions, hCLE is present in complexes where the minimal size correspond to hCLE-hCLE dimers and some of these dimers have a compact structure that is resistant to denaturing conditions.

**Table 1:** Identification of hCLE as monomer and dimer in denaturing gel conditions.

	Protein name	Accesion Number	MW	pI	Pept (MS(MSMS))	Score	Seq.Cov (%)
48 kDa band	hCLE/c14orf166	gi|55613379	28066	6	14 (1)	150	45.4
27 kDa band	hCLE/c14orf166	gi|55613379	28066	6	11	135	42.6

***AC*** **:** Accession Number of the top protein from NCBInr protein Database (non-identical NCBI protein database).

***MW (Mr):*** Nominal molecular weight of each protein.

***pI:*** Calculated pI value.

***Pept (MS[MSMS]):*** number of matched peptides from the top scoring protein in peptide mass fingerprinting and number of MS/MS spectra that were matched to this protein.

***Score:*** Mascot protein score. This number reflects the combined scores of all observed mass spectra that can be matched to amino acid sequences within that protein. A higher score indicates a more confident match.

***Seq Cov:*** Percentage of the database protein sequence covered by matching peptides.

### Characterization of hCLE Associated Proteins

Previous experiments in monkey kidney [1] and human lung epithelial cells [2] showed that hCLE is distributed both in the nucleus and the cytoplasm. In addition, hCLE was isolated from cytoplasmic RNA-containing granules in neurons [8], [9]. Here we tested whether hCLE also has a nuclear and cytoplasmic distribution in the HEK293T human embryonic kidney cells. Furthermore, to gain information about the possible functions of hCLE in the nucleus and the cytosol we characterized its associated proteins.

### Purification of Nuclear and Cytoplasmic hCLE-containing Complexes

**Figure 2:**
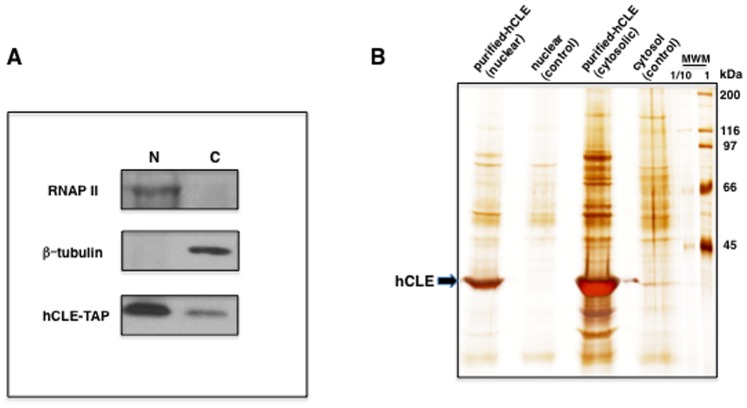
Purification of nuclear and cytosolic hCLE-containing complexes. The hCLE cDNA was inserted upstream of the TAP tag. HEK293T cells were transfected with the hCLE-TAP or the TAP (Control) expressing plasmids and nuclear and cytosolic extracts were used for affinity purification. (A), A sample of the nuclear and cytosolic extracts were analyzed by SDS-PAGE and Western blot in order to confirm the presence of hCLE-TAP protein and to establish the correct subcellular fractioning by detecting a nuclear (RNAP II) and a cytosolic (β-tubulin) protein. (B), Silver stained SDS-PAGE gel of the purified material. The position of hCLE protein is indicated by an arrow. The mobility of molecular weight markers is indicated on the left side of the panel. MWM 1 and 1/10 correspond to 100 and 10 ng of total protein, respectively.

HEK293T cells were transfected with pChCLE-TAP or pC-TAP (control) plasmids and nuclear and cytoplasmic fractions were separated and used for TAP purification. To ensure that subcellular fractionation was correct, the nuclear and cytoplasmic fractions were probed with antibodies that recognize a nuclear (RNA polymerase II), or a cytoplasmic (β-tubulin) protein as well as hCLE-TAP. The results (Fig. 2A) indicated that both subcellular fractions were properly separated and hCLE-TAP was present in both compartments. Next, nuclear and cytoplasmic hCLE and associated purified proteins were obtained and analyzed by SDS-PAGE followed by silver staining (Fig. 2B). Purified hCLE protein was clearly visible and in addition, other protein bands were detected. Nuclear and cytosolic affinity purified samples from hCLE-TAP and TAP expressing cells, were digested with trypsin and the resulting tryptic peptides were analyzed by nano liquid chromatography system coupled to 5600 Triple TOF mass spectrometer, as detailed in Materials and Methods. To estimate the reliability of peptide identification, a FDR (false discovery rate) was calculated at peptide level and peptides with a FDR <1% were considered as statistically significant. Only proteins where at least 2 non-redundant peptides were identified are presented in Table 2 and include proteins that are present exclusively in hCLE purified samples or have at least 3-fold enrichment of total number of confidently identified peptides in hCLE purified fractions compared to the control samples (marked with asterisk). Thus, it corresponds to at least a 3-fold rise in Mascot protein score. The highest Mascot score corresponded to hCLE protein both in nuclear and cytosolic fractions. Table 3 shows the results of hCLE-interacting proteins where only 1 non-redundant peptide was identified. The identification of proteins such as HSPC117, DDX1 and FAM98B that have been previously shown to associate in the same complexes as hCLE [6], [8], [9], [25], validate the utilized approach. Among the proteins found in the hCLE-nuclear complexes, those related with transcription and mRNA processing were the most abundant. The presence of proteins involved in nucleo-cytoplasmic transport and motor-related proteins was also observed. Other proteins involved in cell proliferation and protein translation were detected (Table 3). Regarding the hCLE-interacting proteins in the cytosolic fraction, those functionally related with mRNA transcription, processing and transport were the most abundant. Motor and cytoskeleton-related proteins were also plentiful and the presence of translation proteins was also detected (Table 3). Interestingly, some proteins were associated to hCLE both, in the nucleus and the cytoplasm such as DDX1, HSPC117, FAM98B or myosin light chain kinase 2.

**Table 2:** Nuclear and cytosolic hCLE-associated proteins.

Nuclear hCLE-associated proteins								
		Protein Name	Accession Number UniProtKB /Swiss-Prot	MW (Da)	Identified Pep (non-redundant) hCLE-CBD	Score hCLE-CBD	Identified Pep (non-redundant) Ctr.	Score Ctr.
**Transcription factors and** **related proteins**		*ATP-dependent RNAhelicase DDX1	Q92499	83162	41(33)	1766	9(7)	304
		*RTCB_HUMAN tRNA-splicing ligase (HSPC 117)	Q9Y3I0	55589	39(35)	1666	6(6)	248
		*Protein FAM98B	Q52LJ0	37489	13(13)	655	3(2)	203
		*Single-stranded DNA-binding protein 3*	Q9BWW4	40533	2(2)	131	–	–
		*Transcription factor 4*	P15884	71448	2(2)	120	–	–
		*Serrate RNA effector molecule homolog*	Q9BXP5	10092	2(2)	82	–	–
		*Heterogeneous nuclear ribonucleoprotein R*	O43390	71129	2(2)	72	–	–
		*Polyadenylate-binding protein 1 (PABP1)*	P11940	70810	2(2)	61	–	–
		*YTH domain family protein 3*	Q7Z739	63914	2(2)	59	–	–
**Nucleo-cytoplasmic transport and cytoskeleton-related factors**		*Myosin light chain kinase 2, skeletal/cardiac muscle	Q9H1R3	65104	5(2)	193	1(1)	61
		*Myosin light polypeptide 6*	P60660	17057	2(2)	93	–	–
		*ZW10 interactor*	O95229	31320	2(2)	63	–	–
		*Ran GTPase-activating protein 1*	P46060	63870	2(2)	61	–	–
		*Liprin-alpha-1*	Q13136	13615	2(2)	61	–	–
**Nuclear structural proteins**		*Nuclear pore complex protein Nup153	P49790	15511	4(4)	208	1(1)	52
**Others**		*Protein FAM98A*	Q8NCA5	55734	14(12)	707	–	–
		*Nibrin*	O60934	85458	3(3)	119	–	–
		*protein C14orf166 (CLE)	Q9Y224	28143	78(21)	3899	10(10)	513
**Cytosolic hCLE-associated Proteins**								
		**Protein name**	**Accession Number UniProtKB/Swiss-Prot**	**MW** **(Da)**	**Identified Pep (non-redundant) hCLE-CBD**	**Score hCLE-CBD**	**Identified Pep (non-redundant) Ctr.**	**Score Ctr.**
**Transcription factors and related proteins**	*ATP-dependent RNA helicase DDX1		Q92499	83162	86(49)	3543	11(11)	423
	*RTCB_HUMAN tRNA-splicing ligase (HSPC 117)		Q9Y3I0	55589	64(35)	3072	8 (7)	234
	*Protein FAM98B		Q52LJ0	37489	34(18)	1570	3(3)	167
	*Putative RNA binding protein Luc7-like 1*		Q9NQ29	44023	2(2)	123	–	–
**Transport and cytoskeleton related factors**	*Myosin light chain kinase 2, skeletal/cardiac muscle		Q9H1R3	65104	21(2)	716	6(2)	219
	*ATP-binding cassette sub-family D member 3*		P28288	75841	6(6)	243	–	–
	*TATA-binding protein-associated factor 2N*		Q92804	61977	3(3)	158	–	–
	*B-cell receptor-associated protein 31*		P51572	28020	4(4)	149	–	–
**Chaperons**	*DnaJ homolog subfamily C member 11*		Q9NVH1	63469	3(2)	119	–	–
	*DnaJ homolog subfamily B member 6*		O75190	36111	2(2)	58	–	–
**Others**	*Golgin subfamily A member 3		Q08378	167666	137(98)	6784	1(1)	72
	*Protein FAM98A		Q8NCA5	55734	20(13)	818	3(3)	183
	*Protein FAM98C*		Q17RN3	37903	5(5)	256	–	–
	*Ubiquitin-40S ribosomal protein S27a		P62979	18229	9(5)	447	3(3)	117
	*Coiled-coil domain-containing protein 8*		Q9H0W5	59431	2(2)	82	–	–
	*protein C14orf166 (CLE)		Q9Y224	28143	591(46)	34290	14(13)	698

Table includes proteins where at least 2 non-redundant peptides were identified, with a FDR <1% at peptide level that are present exclusively in hCLE purified samples or have at least 3-fold enrichment of total number of confidently identified peptides in hCLE purified fractions compared to the control (ctr.) samples (marked with asterisk). Their molecular weight, the total number of confidently identified peptides and the number of non-redundant ones, as well as the protein Mascot scores, is shown.

**Table 3:** Nuclear and cytosolic hCLE-associated proteins.

Nuclear hCLE-associated proteins										
	Protein Name	Accession Number UniProtKB/Swiss-Prot		MW (Da)		Identified Pep (non-redundant) hCLE-CBD		Score hCLE-CBD		
**Transcription factors and related proteins**	*5∼-3∼exoribonuclease 2*	Q9H0D6		109249		2(1)		70		
	*Heterogeneous nuclear ribonucleoprotein A3 (hnRNP A3)*	P51991		39755		1(1)		67		
	*F-box-like/WD repeat-containing protein*	Q9BQ87		57158		1(1)		64		
	*Transcription elongation factor SPT6 (SPT6)*	Q7KZ85		199960		1(1)		60		
	*Heterogeneous nuclear ribonucleoprotein DO (hnRNP DO)*	Q14103		38548		1(1)		59		
	*Heterogeneous nuclear ribonucleoprotein C1/C2*	P07910		33696		1(1)		51		
	*AT-rich interactive domain-containing protein 3A (ARI3A)*	Q99856		62896		1(1)		49		
	*LIM domain-binding protein 1*	Q86U70		47008		1(1)		46		
**Splicing factors**	*Pre-mRNA 3∼-end-processing factor FIP1*	Q6UN15		66579		1(1)		93		
	*Pre-mRNA-processing factor 19*	Q9UMS4		55514		1(1)		71		
	*Small nuc. ribonucleoprotein-associated proteins B and B∼*	P14678		24732		1(1)		55		
	*Pre-mRNA-processing-splicing factor 8*	Q6P2Q9		274484		1(1)		49		
	*RNA-binding protein 25*	P49756		100400		1(1)		46		
	*U4/U6 small nuclear ribonucleoprotein Prp4*	O43172		58964		1(1)		45		
	*Splicing factor 3B subunit 2*	Q13435		100257		1(1)		43		
**Motor proteins and cytoskeleton-related factors**	*Kinesin-like protein KIF20A*	O95235		101043		1(1)		106		
	*Kinesin-like protein KIF2A*	O00139		80456		3(1)		89		
	*Myosin-9*	P35579		227403		2(1)		56		
	*signal-induced proliferation-associated 1-like protein 1*	O43166		200870		1(1)		57		
	*Spectrin beta chain, non-erythrocytic 5*	Q9NRC6		418740		2(1)		57		
**Nuclear structural proteins**	*Lamin-B receptor*	Q14739		70980		1(1)		52		
	*Emerin*	P50402		29022		1(1)		51		
**Proliferation**	*Coiled-coil alpha-helical rod protein 1*	Q8TD31		88847		1(1)		75		
	*Signal-induced proliferation-associated 1-like protein 2*	Q9P2F8		191974		1(1)		75		
	*Nucleus accumbens-associated protein 1*	Q96RE7		57774		1(1)		62		
	*Suprabasin*	Q6UWP8		25366		1(1)		59		
	*Protein salvador homolog 1*	Q9H4B6		44698		1(1)		54		
	*Vasodilator-stimulated phosphoprotein*	P50552		39943		1(1)		48		
	*Serine/threonine-protein kinase WNK1*	Q9H4A3		251376		1(1)		44		
	*Small proline-rich protein 3*	Q9UBC9		18510		1(1)		43		
**DNA metabolism**	*DNA replication licensing factor MCM7*	P33993		81762		1(1)		87		
**Translation**	*60S ribosomal protein L10-like*	Q96L21		24871		1(1)		43		
	*60S acidic ribosomal protein P2*	P05387		11658		1(1)		43		
	*40S ribosomal protein S30*	P62861		6644		1(1)		42		
**Others**	*E3 SUMO-protein ligase PIAS1*	O75925		72572		1(1)		99		
	*Lysozyme C*	P61626		16894		1(1)		81		
	*L-lactate dehydrogenase A chain*	P00338		36895		1(1)		64		
	*S-adenosylmethionine synthase isoform type-2*	P31153		43909		1(1)		55		
	*DET1- and DDB1-associated protein 1*	Q9BW61		11874		1(1)		53		
**Cytosolic hCLE-associated Proteins**										
	**Protein name**		**Accession Number UniProtKB/Swiss-Prot**		**MW (Da)**		**Identified Pep (non-redundant) hCLE-CBD**		**Score hCLE-CBD**	
**Transcription factors and RNA processing**	*Transcriptional repressor p66-beta*		Q8WXI9		65496		1(1)		83	
	*Ribosome biogenesis protein BMS1 homolog*		Q14692		146406		1(1)		72	
	*Ribosomal biogenesis protein LAS1L*		Q9Y4W2		83795		1(1)		44	
	*Bystin*		Q13895		49754		1(1)		43	
**Transport and cytoskeleton-related factors**	*Potassium-transporting ATPase alpha chain 1*		P20648		115425		1(1)		76	
	*Syntaxin-18*		Q9P2W9		38788		1(1)		67	
	*Spectrin beta chain, non-erythrocytic 5*		Q9NRC6		418740		2(1)		57	
	*Rab11 family-interacting protein 5*		Q9BXF6		70556		1(1)		52	
	*ATP-binding cassette sub-family F member 2*		Q9UG63		71704		1(1)		52	
	*Fragile X mental retardation-interacting protein 2*		Q7Z417		76121		1(1)		45	
	*Tight junction protein ZO-2*		Q9UDY2		134060		1(1)		44	
	*Microfibrillar-associated protein 1*		P55081		51927		1(1)		42	
	*Emerin*		P50402		29022		1(1)		37	
	*Pericentrin*		O95613		380059		1(1)		35	
**Proliferation**	*Disintegrin and metalloproteinase domain-containing prot. 9*		Q13443		92521		1(1)		48	
	*Migration and invasion-inhibitory protein*		Q5JXC2		43257		1(1)		35	
**Translation**	*40S ribosomal protein S15*		P62841		17029		1(1)		78	
	*Putative 60S ribosomal protein L13a-like MGC87657*		Q6NVV1		12173		2(1)		68	

Table includes proteins where 1 non-redundant peptides were identified, with a FDR <1% at peptide level that are present exclusively ihCLE purified samples. Their molecular weight, the total number of confidently identified peptides and the number of non-redundant ones, as well as the protein Mascot scores, is shown.

### Purified hCLE Associates with RNAs

**Figure 3:**
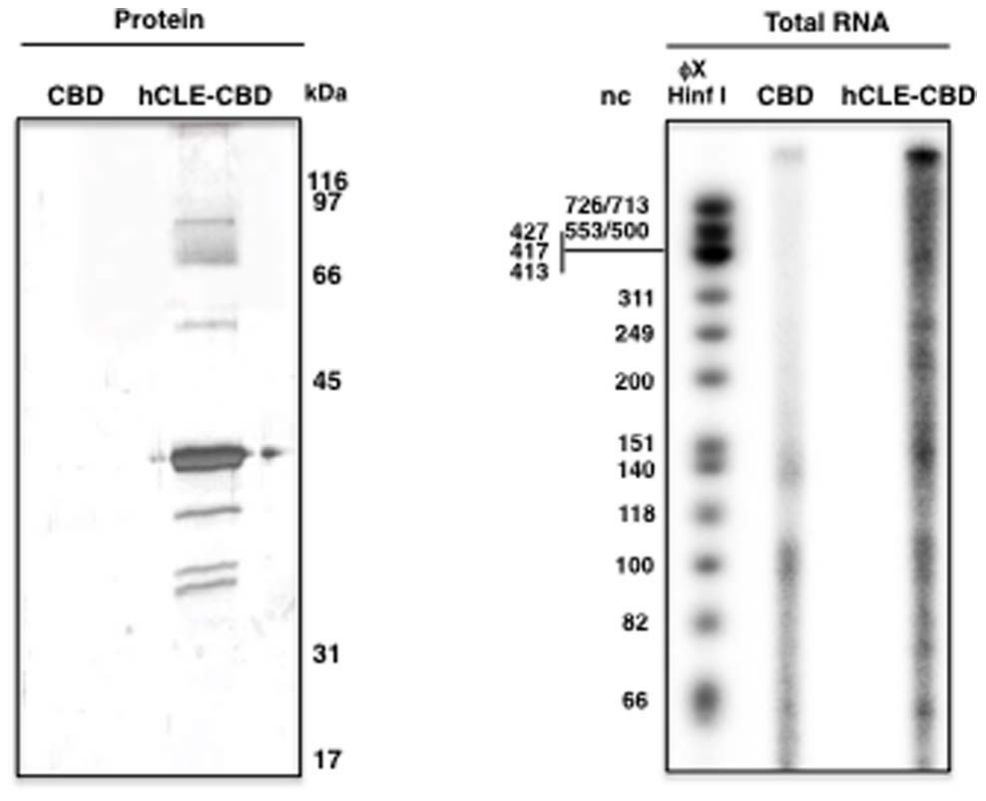
RNA analysis. HEK293T cells were transfected with control pC-TAP or pChCLE-TAP plasmids and the RNA associated to CBD or hCLE-CBD after DNAse treatment was isolated from the purified complexes, radiolabeled and analyzed in denaturing acrylamide-urea 15% gels. (A); protein silver staining. (B); Radiolabeled RNA.

The above results together with biochemical approaches [5], [7], [8], [9] indicate that hCLE is a component of large complexes, which should have associated RNAs. To examine that we analyzed the presence of RNAs in the purified hCLE-protein complexes treated with DNAse. RNAs were isolated from control and hCLE-CBD purified protein from total cell extracts, labeled as described in Materials and Methods and separated in SDS-urea PAGE gels. A clear enrichment of RNAs corresponding to different sizes was observed in hCLE samples compared to control (Fig. 3). These results are in agreement with the presence of hCLE in RNAP II-directed RNA transcription sites [3] and with a recent report describing hCLE as an RNA-binding protein [26] and therefore suggest that RNA could play a role mediating the composition of hCLE-complexes.

### Nuclear and Cytoplasmic Purified hCLE-interacting Proteins are Present in Complexes with the Endogenous hCLE

**Figure 4:**
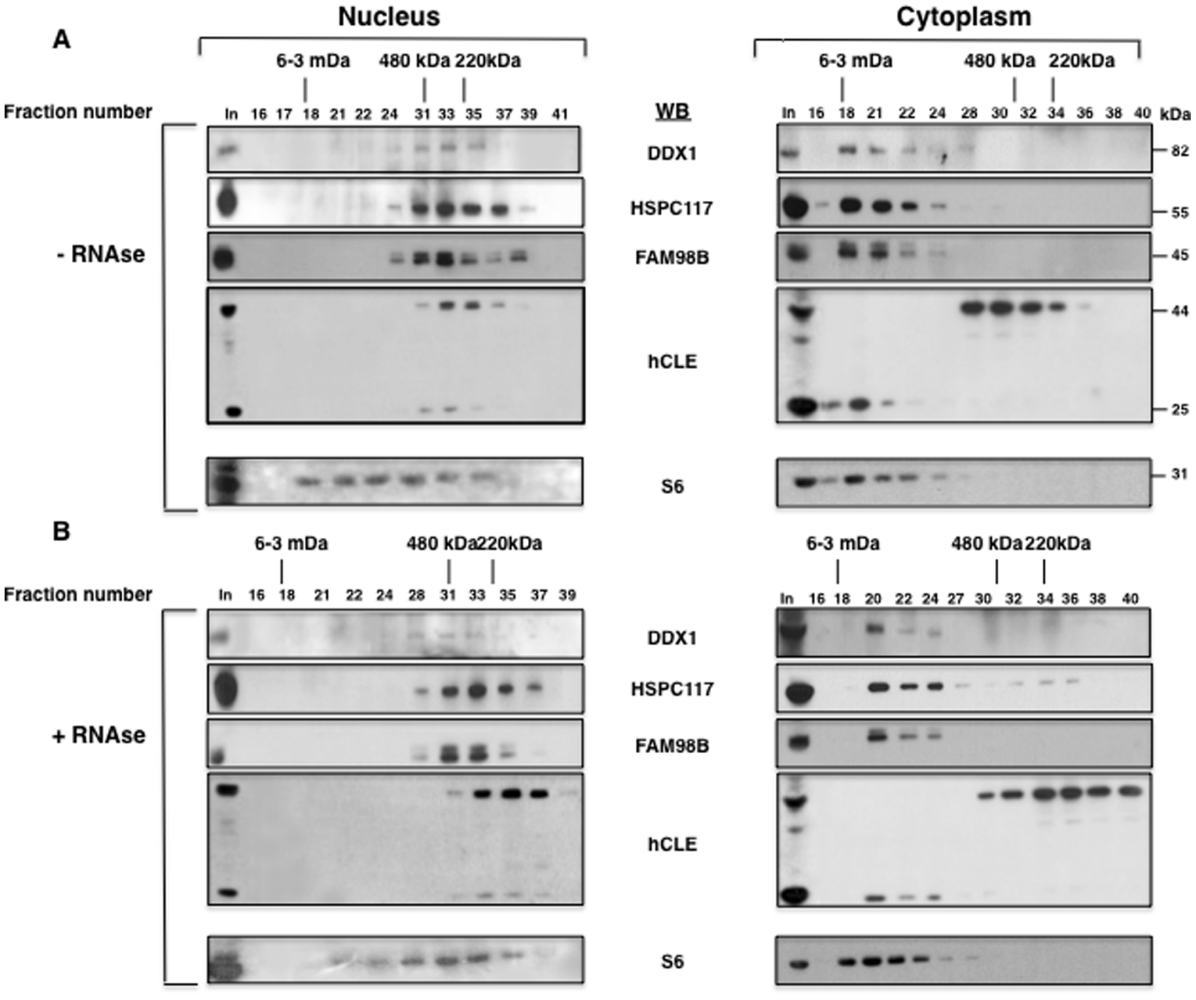
Gel filtration separation of the hCLE-containing complexes. (A); HEK293T nuclear or cytosolic cell extracts were untreated (A) or treated (B) with RNAse A and filtered through a Sephacryl S400 column and their elution was monitored by Western blot against the indicated proteins. The lines denote the elution of the MW markers.

The proteomic data indicated that purified hCLE is a component of protein complexes and also that the same complexes are found in both, the nucleus and the cytoplasm. To examine whether the endogenous hCLE is also present in protein complexes and to test the presence of those purified hCLE-interacting nuclear and cytosolic proteins, we prepared nuclear and cytosolic extracts of HEK293T cells and analyzed them by S-400 molecular exclusion chromatography column. As described above, purified hCLE-complexes contain RNAs and a large number of hCLE-interacting proteins are involved in RNA metabolism and moreover some of them have been characterized as RNA-binding proteins such as DDX1 and hCLE itself [26]. Therefore, we wondered whether RNA would be involved in the formation of endogenous hCLE complexes and thus the cell extracts were both untreated or treated with RNAse before being used for S-400 molecular exclusion chromatography. The collected fractions were analyzed by Western blot with anti-hCLE antibodies and with antibodies that recognize some of the proteins that appear associated with hCLE in both subcellular compartments. The examination of the nuclear extracts indicated that in the absence of RNAse treatment, hCLE is present in protein complexes of 220–480 kDa or smaller together with DDX1, HSPC117 and FAM98B (Fig. 4A left). Treatment with RNAse of the nuclear extracts produced a decrease in the size of the hCLE complexes, which are smaller than 220 kDa and contain a portion of HSPC117. In these conditions, most of DDX1, HSPC117 and FAM98B remained together in complexes of around 220–480 kDa (Fig. 4B left). On the other hand, both hCLE monomers and dimers fractioned equally in untreated or RNAse treated extracts. Therefore, endogenous nuclear hCLE seems to associate to those proteins bound to the purified protein, and the association is at least in part RNA mediated.

Next we analyzed the behavior of the cytosolic endogenous hCLE protein in untreated or RNAse-treated cells. In this case hCLE from untreated extracts is present in two types of protein complexes; a large complex together with DDX1, HSPC117 and FAM98A of mDa where hCLE is not resistant to denaturing conditions and therefore migrates as a monomer and in a second peak larger than 480 kDa which is free of the accompanying proteins and where hCLE is exclusively present as dimer (Fig. 4A right). The RNAse treatment produces a decrease in the size of these two complexes but not in the composition, since monomer of hCLE, DDX1, HSPC117 and FAM98A are still present in the bigger complexes and absent in the smaller ones containing hCLE dimers (Fig. 4B right). Therefore these results indicate that RNA is present in hCLE cytosolic complexes, but the association of cytosolic hCLE to the other characterized proteins is RNAse resistant and involves protein-protein interactions. In addition they indicate that the hCLE form that is resistant to denaturing condition is not associated to DDX1, HSPC117 and FAM98B but to some other proteins found in the proteomic analysis.

The proteomic data indicated the existence of ribosomal proteins of the 40 and 60S subunits associated with purified hCLE as well as cytoskeleton-related proteins (Table 3). To examine the distribution of ribosomal proteins with the different complexes of the endogenous hCLE, the above fractions were subjected to Western blot analysis to detect total ribosomal protein S6. The nucleo-cytoplasmic shuttling of S6 protein has been extensively characterized. Following translation in the cytoplasm, S6 is targeted to the nucleolus where it assembles into 40S preribosomal subunits. Nascent subunits are translocated to the nucleoplasm, what induces a specific ribosomal maturation pathway. Mature preribosomal subunits shuttle back to the cytoplasm and finally fuse with 60S ribosomal subunits to form mature, functional ribosomes [27], [28]. In addition, S6 protein has been detected in the dendritic RNA granules that transport specific mRNAs [29]. Previous publications reported the presence of hCLE in these cytosolic RNA granules transporting specific mRNAs which contain RNA-binding proteins, ribosomes and microtubule-associated proteins and whose local translation in neuronal processes plays a crucial role in developing brain [9]. Analysis of S6 distribution in the nucleus showed that a portion of the protein fractionates with hCLE and DDX1, HSPC117 and FAM98B and under RNAse treatment, S6 remains in complexes of 220–480 kDa while hCLE is mainly in complexes of around 220 kDa. These results indicate that S6 may associate with hCLE-DDX1-HSPC117-FAM98B complexes in an RNA-dependent manner. Looking at the S6 cytosolic distribution, we observed that its migration strictly correlates with the presence of hCLE as monomer in the presence or absence of RNAse treatment, together with DDX1, HSPC117 and FAM98B, supporting the existence of a complex with these proteins.

**Figure 5:**
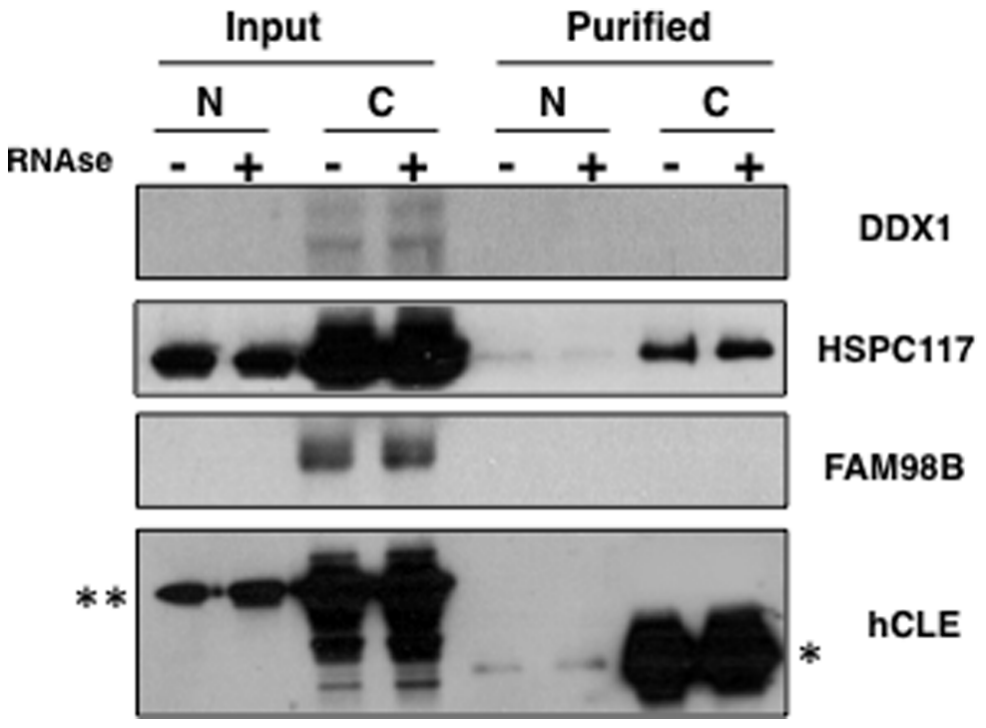
Effect of RNAse treatment in the association of purified hCLE-associated proteins. Cultured HEK293T cells were transfected with pChCLE-TAP plasmid, nuclear (N) and cytosolic (C) cell extracts were untreated or treated with RNAse A and used for hCLE purification. The presence of DDX1, HSPC117, FAM98B and hCLE in the original extracts (input) or the purified fraction (purified) was monitored by Western blot. (**); denotes the hCLE-TAP protein, (*), denotes the hCLC-CBD purifed protein.

Since purified hCLE associates in the nucleus and the cytoplasm with DDX1-HSPC117-FAM98B and the purified complexes contain RNAs (Fig. 3), we examined the effect of RNAse treatment in these associations. Nuclear and cytosolic fractions of pChCLE-TAP HEK293T transfected cells, were treated or not with RNAse, hCLE-TAP together with its associated proteins was purified and the presence of DDX1, HSPC117 and FAM98B was analyzed by Western blot (Fig. 5). The overexpressed hCLE-TAP (**) and endogenous HSPC117 were clearly detected in the nuclear and cytoplasmic fractions of the cell extracts (Input), whereas DDX1 and FAM98B were only detected in the cytoplasmic fraction and at much lesser extent than HSPC117. As a consequence, the analysis of the purified fraction only allowed the detection of purified hCLE-CBD (*) and HSPC117 proteins. RNAse treatment of nuclear extracts produced a partial dissociation of hCLE-CBD and HSPC117 similarly to the observed dissociation of endogenous hCLE (Fig. 4). By contrast RNAse treatment of the cytosolic fraction did not change this association. Therefore, purified hCLE behaves similarly to endogenous hCLE and in both cases it seems that the nuclear association of hCLE to HSPC117 is, at least in part, RNA mediated whereas the cytosolic interactions seems to be mediated by protein-protein interactions.

### hCLE Modulates the Expression of hCLE-interacting Proteins

**Figure 6:**
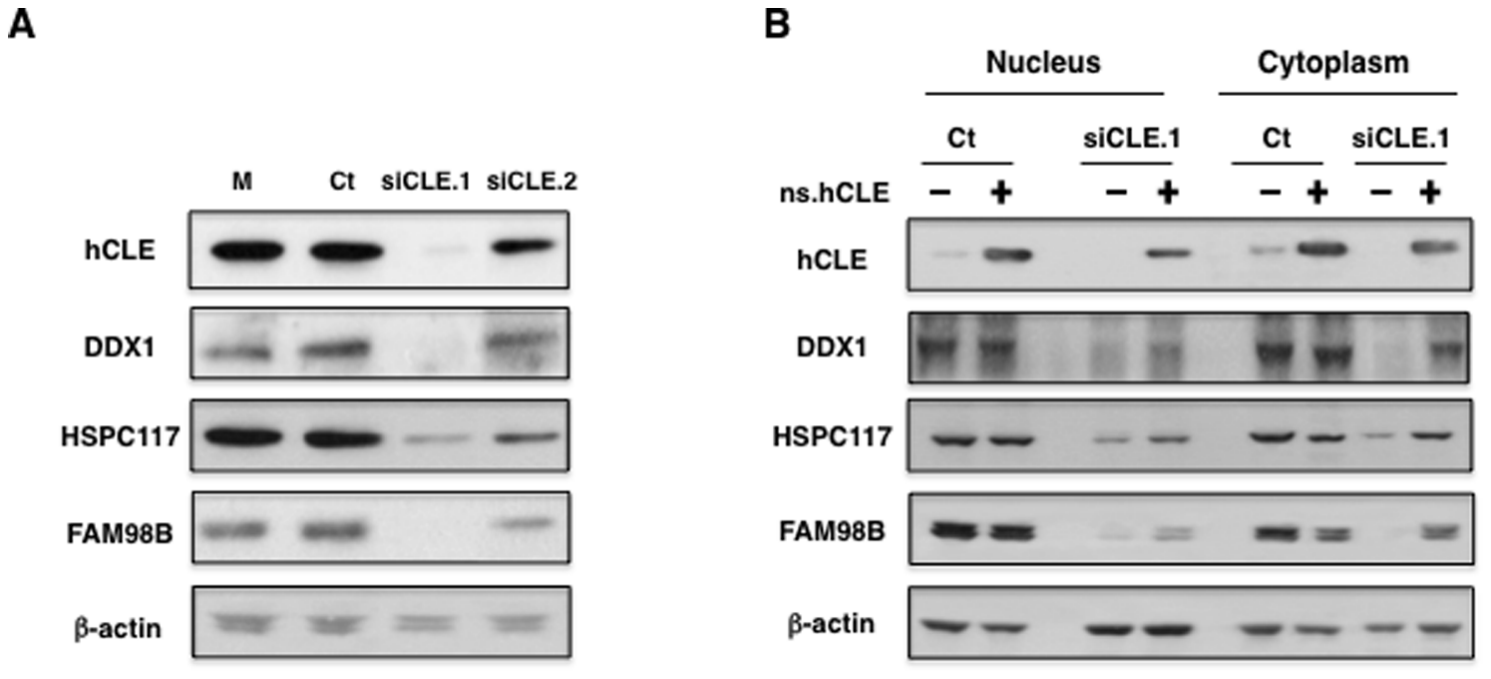
hCLE modulates the expression of hCLE-interacting proteins. (A); HEK293T cells were infected with lentiviruses expressing a control sequence (Ct) or specific sequences for hCLE silencing (siCLE.1 and si-CLE.2) and 4 days post-infection total cell extracts were used for Western blot against the indicated proteins. (B); HEK293T cells were infected with lentiviruses expressing a control sequence (Ct) or a specific sequence for hCLE silencing (siCLE.1). 5 days post-infection, the cells were left untransfected (-) or transfected (+) with a plasmid expressing a hCLE gene with 3 silent mutations that avoids its silencing (ns.hCLE). 48****h post-transfection, nuclear and cytoplasmic fractions were prepared and used for Western blot detection of the indicated proteins.

Next approach was to evaluate whether hCLE modulates the expression of the other components of the hCLE-DDX1-HSPC117-FAM98B complexes. Previous silencing experiments in HeLa cells indicated that depletion of HSPC117, the essential subunit of a human tRNA splicing ligase complex, which contains HSPC117, DDX1, hCLE, FAM98B and ASW proteins [6] provoked the down-regulation of hCLE, DDX1 and FAM98B. In contrast, depletion of hCLE did not down-regulate HSPC117 expression but did down-regulate DDX1 and FAM98B. Thus, to examine the effect of hCLE down-regulation in HEK293T, the cells were infected with lentiviruses expressing a control siRNA or siRNAs that target two different regions of hCLE and total cell extracts from the infected cells were used for Western blots against hCLE, DDX1, HSPC117, FAM98B and β-actin as control. Depletion of hCLE caused the down-regulation of the hCLE-interacting proteins without altering β-actin content (Fig. 6A). Moreover, to study the possible recovery of hCLE-interacting proteins by hCLE overexpression, control or hCLE silenced cells were transfected with a plasmid that expresses a non-silenceable hCLE gene, and nuclear and cytoplasmic fractions were obtained and tested for recovery of the down-regulated proteins. The results (Fig. 6B) showed that hCLE silencing down-regulates the nuclear and cytoplasmic expression of HSPC117, FAM98B and DDX1 and its overexpression recovers the levels of these proteins in both subcellular compartments. The data suggest that down-regulation of hCLE provokes destabilization of the hCLE-DDX1-HSPC117-FAM98B complex and consequently degradation of the associated proteins, and indicates that regulation of these nuclear and cytoplasmic complexes is very tightly linked, which suggests a prominent functional interaction. Similar results have been reported for protein complexes in different systems such as the small γ-tubulin complex, the major component of the pericentriolar material [30], or the mammalian HRD1-SEL1L complex, which provides a scaffold for endoplasmic reticulum associated degradation [31].

### HCLE is a Shuttling Protein

**Figure 7:**
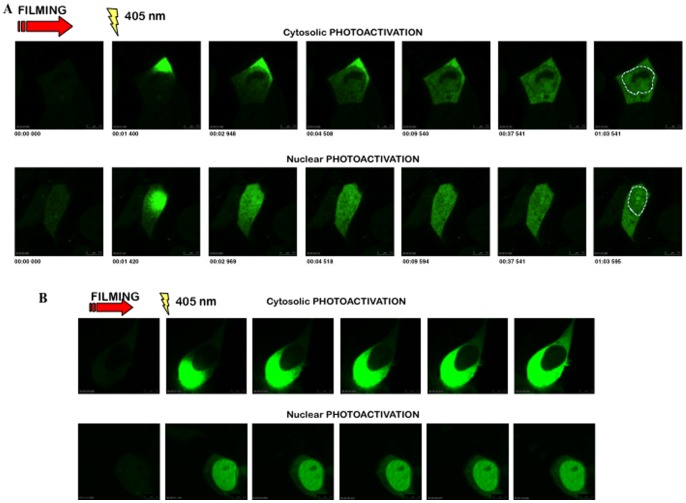
hCLE shuttles in and out of the nucleus. (A); Cultured HEK293T cells were transfected with the phCLE-PAGFP (photoactivatable GFP) plasmid and 24 h post-transfection they were used for live cell microscopy. The videos were analyzed with Leica Microsystems LAS AF software (version 2.3.0). (upper panel), Photoactivation was applied in the cytosol to visualize hCLE import. (lower panel), Photoactivation was applied in the nucleus to visualize hCLE export. The numbers below the figures represent minutes, seconds and milliseconds after photoactivation. A dotted line marking the boundary of the nucleus is included in the last panels. (B); Cultured HEK293T cells were transfected with the empty pPAGFP plasmid and 24 h post-transfection they were used for live cell microscopy. Photoactivation was applied in the nucleus and the cytosol to visualize GFP movement.

Since hCLE is associated to several proteins that are common in the nucleus and the cytosol we considered two possible scenarios: either hCLE is a nucleo-cytoplasmic shuttling protein that moves in complexes together with its associated proteins, or alternatively two different hCLE populations exist in the cell that localize in the nucleus or in the cytoplasm. To examine that we prepared a construct that expresses hCLE tagged with a photoactivatable GFP (PAGFP) [15] which has a particularly dim fluorescence if not photoactivated, but when excited at a wavelength of 405 nm, gives a measurable peak of emission at 488 nm. HEK293T cells were transfected with the phCLE-PAGFP plasmid and 24 h thereafter were used in photoactivation and live cell microscopy experiments. Photoactivation in the cytoplasm allowed us to detect the import of hCLE into the nucleus (Fig. 7A, upper panel), whereas photoactivation in the nucleus proved that hCLE is also exported from the nucleus to the cytoplasm (Fig. 7A, lower panel). hCLE enters the nucleus slower than it leaves it, as can be seen in the representative examples where at 2 s and 969 ms hCLE has exited the nucleus after its photoactivation, while entering the nucleus after cytoplasmic photoactivation takes 9 s and 540 ms. As control we performed photoactivation of GFP in the nucleus and the cytoplasm and, as can be seen, it remains in these compartments after photoactivation (Fig. 7B). These results enabled us to establish that hCLE is a novel shuttling protein, with different kinetics for its nucleo-cytoplasmic movement (compare the corresponding videos S1 and S2).

### HCLE Import is Dependent on Active Transcription

**Figure 8:**
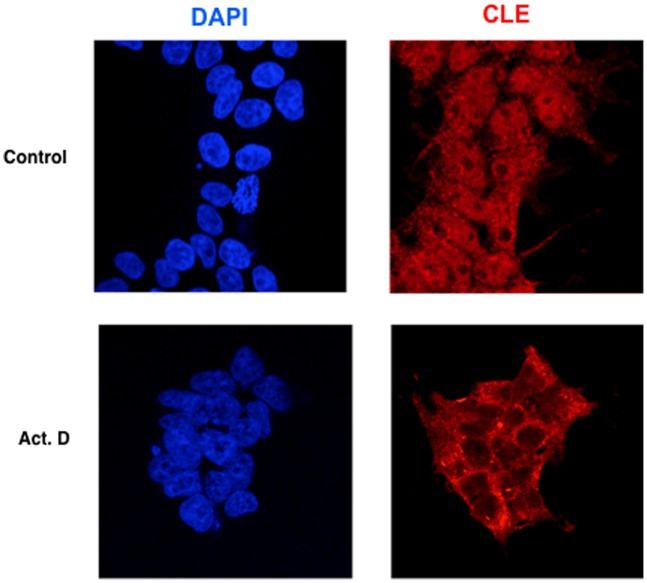
Transcription inhibition retains hCLE in the cytoplasm. Cultured HEK293T cells were incubated in the absence (Control) or the presence of Actinomycin D (Act. D) during 1 h, washed, fixed and processed for immunofluorescence using antibodies anti-hCLE and DAPI.

**Figure 9:**
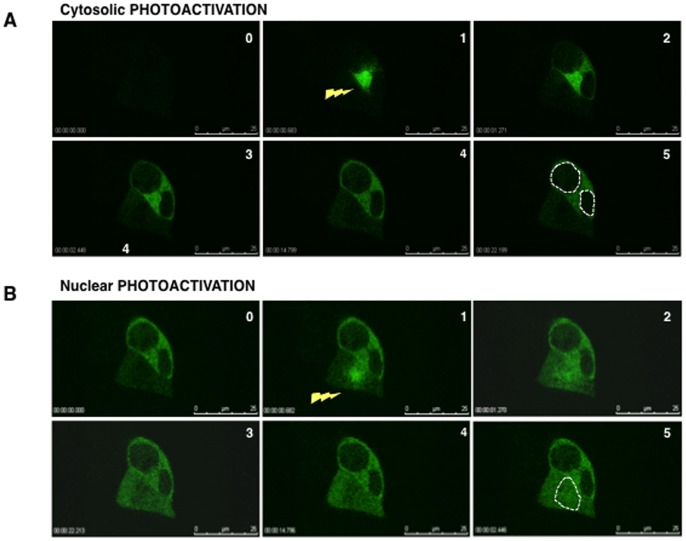
hCLE nuclear import is transcription dependent. Cultured HEK293T cells were transfected with the phCLE-PAGFP (photoactivatable GFP) plasmid and 24 h post-transfection they were treated with Actinomycin D during a 10 min pulse, washed and used for live cell microscopy. (A), Photoactivation was applied in the cytosol to visualize hCLE import. (B), Photoactivation was applied in the nucleus to visualize hCLE export. A dotted line marking the boundary of the nucleus is included in the last panels.

As described above, hCLE seems to be closely related with nuclear and cytoplasmic RNA metabolism. The nuclear export of the majority of mRNAs is coupled to ongoing gene transcription in mammalian cells [32] therefore, it is conceivable that the subcellular distribution of the RNA-binding proteins that may be involved in mRNA transport would be dependent on active transcription. To analyze if this was the case with hCLE, HEK293T cells were treated with Actinomycin D, a general transcription inhibitor and the localization of hCLE was examined by immunofluorescence. Treatment with Actinomycin D clearly increased the cytoplasmic localization of hCLE suggesting that inhibition of RNA transcription prevents the nuclear entrance of hCLE (Fig. 8). To confirm the prevention of hCLE nuclear entrance in Actinomycin D treated cells, HEK293T cells were transfected with the plasmid that expresses the photoactivatable hCLE, treated with Actinomycin D and used in photoactivation and live cell microscopy experiments. Transcription inhibition did not disturb the export of hCLE from the nucleus (Fig. 9B), whereas it totally impaired its nuclear import (Fig. 9A), (compare the corresponding videos S3 and S4).

### The Import of hCLE-associated Proteins is also Dependent on Active Transcription

**Figure 10:**
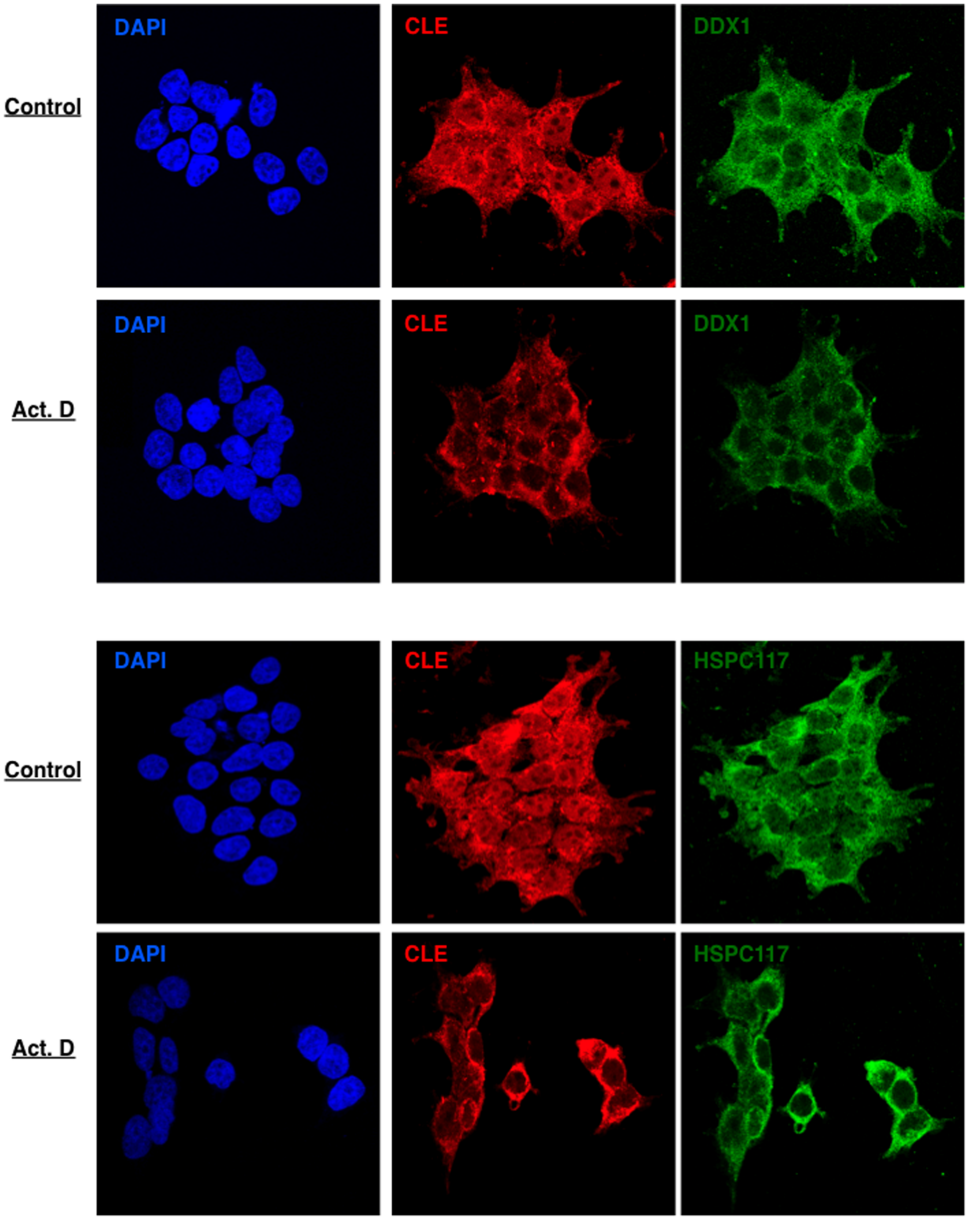
Transcription inhibition retains DDX1 and HSPC117 in the cytoplasm. Cultured HEK293T cells were incubated in the absence (Control) or the presence of Actynomicin D (Act. D) during 30 min, washed, fixed and processed for immunofluorescence using antibodies anti-hCLE, DDX1, HSPC117 and DAPI.

**Figure 11:**
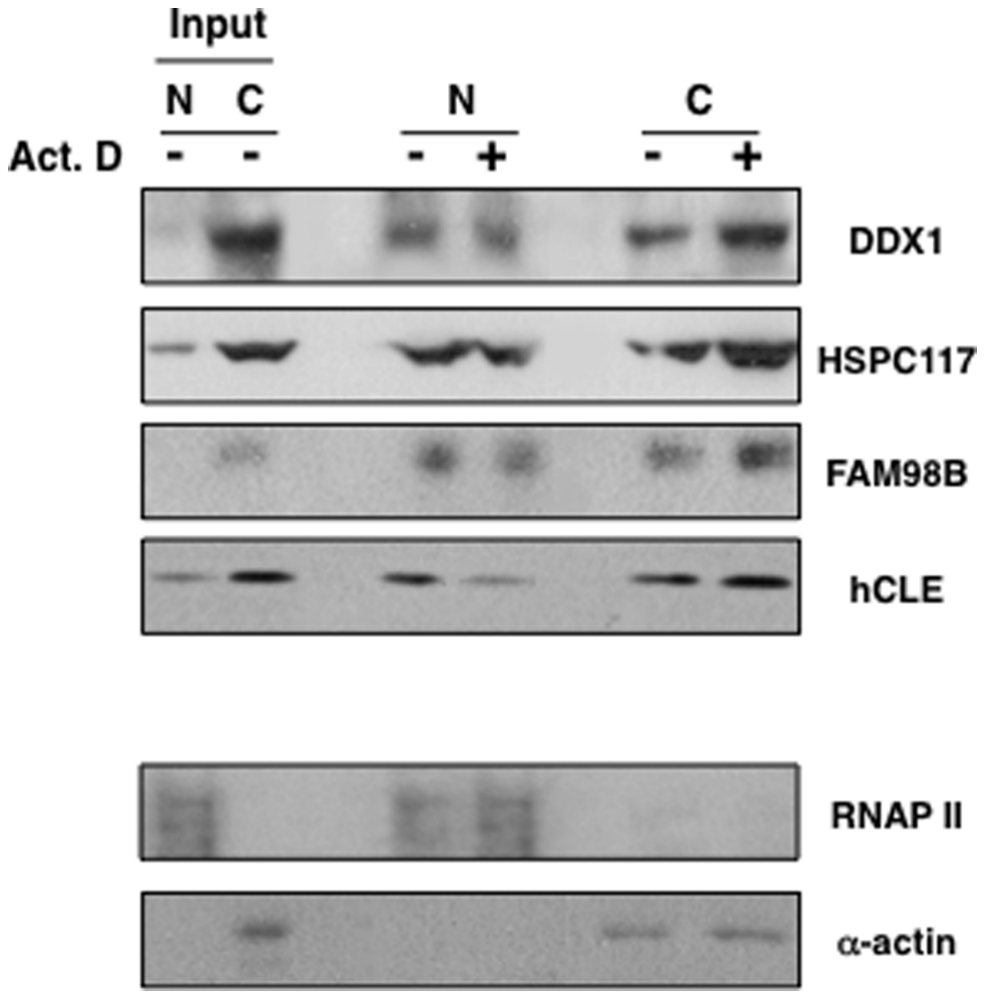
Transcription inhibition increases the cytosolic accumulation of hCLE, DDX1, HSPC117 and FAM98B. Cultured HEK293T cells were incubated in the absence or the presence of Actinomycin D (Act. D) during 1 h. Nuclear and cytoslic extracts were prepared and used for Western blot assays to detect the indicated proteins. (N); nuclear fraction, (C), cytosolic fraction. RNAP II and α-actin were used as markers for subcellular fractionation.

Since hCLE is closely associated to DDX1, HSPC117 and FAM98B we examined whether the nucleo-cytoplasmic distribution of these hCLE-interacting proteins was also dependent on active transcription. Control or Actinomycin D HEK293T-treated cells were processed for immunofluorescence analysis to detect HSPC117 and DDX1 distribution besides hCLE localization (FAM98B could not be examined due to the absence of proper antibodies) (Fig. 10). Both, HSPC117 and DDX1 present a more prominent cytoplasmic than nuclear distribution, but treatment with Actinomycin D produces a clear retention of these two proteins in the cytoplasm in parallel with hCLE behavior, reinforcing the functional association of the complexes. To confirm these results we performed nuclear and cytosolic separation of untreated or Actinomycin D treated cells at 5 µg/ml during 1 h, and analyzed the presence of hCLE, DDX1, HSPC117 and FAM98B in both types of fractions as well as RNAP II and α-actin as nuclear and cytosolic markers, respectively (Fig. 11). Using similar amounts of nuclear and cytosolic fractions, in terms of cell number per volume of extraction represented in the Input, it can be observed that hCLE, DDX1, HSPC117 and FAM98B are mainly present in the cytosol. Thus the analysis of Actimomycin D on protein distribution was performed using high amounts of the nuclear fraction. The results indicated that treatment with Actinomycin D produces a decrease of these proteins in the nuclear compartment together with an increase in the cytosolic fraction. Collectively these results indicate that active transcription is required to permit nuclear localization of hCLE, probably in protein complexes with DDX1-HSPC117-FAM98B, and suggest that synthesis of new RNA cargos are required to trigger hCLE-complex nuclear import.

## Discussion

The biology of RNA is orchestrated by the interplay of RNAs with RNA-binding proteins within dynamic ribonucleoproteins (RNPs). The RNPs direct and coordinate the destiny of the transported RNAs which may present an asymmetric distribution since some of them will be degraded, translated, stored in different types of RNA granules or even localized in specific subcellular sites to be translated locally in response to specific stimuli. High-throughput approaches have revealed that 70% of mRNA species are asymmetrically distributed [33] and a very large number of proteins participate in the process, although the molecular mechanisms responsible for the coupling of post-transcriptional regulatory networks are yet poorly understood. A finding that has become commonplace is the presence of established nuclear proteins in cytosolic RNP complexes, indicating that there are nuclear and cytoplasmic steps in the RNA localization pathway, and suggesting that the binding of specific RNA-binding proteins in the nucleus may play a role in the fate of RNA in the cytoplasm. A plausible hypothesis is that these RNA-binding proteins could link cytoplasmic RNA localization to earlier nuclear events in RNA biogenesis such as transcription and splicing.

### hCLE takes Part in RNPs Involved in RNA Metabolism

One of the factors that may play a role in the life of RNA is hCLE, that not only has been repeatedly found as a core component of different cytosolic RNA transporting granules [8], [9], [25], but also has been shown to be part of the spliceosome [4] and the human tRNA splicing ligase complex [6], to be an interactor of the capping enzyme [5] and to positively modulate the RNAP II-derived transcription [3]. All these features point to an important role of hCLE in the complex life of RNAs, and thus we have tried to elucidate how hCLE participates in RNA metabolism, both as a nuclear factor and as a potentially important actor in the successive cytosolic stages. If that is the case, hCLE should be associated with nuclear and cytoplasmic proteins involved in nuclear events such as transcription, processing and transport and cytosolic events as translation, since these interactions would be a requirement for the proposed function of hCLE in the fate of the RNA.

Regarding the nuclear interactions that hCLE establishes with factors involved in the synthesis and processing of RNAs, we have found transcription factor 4; a protein member of the basic helix-loop-helix transcription factor family whose mutation has been identified as involved in the Pitt-Hopkins disorder [34], hnRNP R; which functions as a transcriptional coactivator of the c-fos promoter [35], or PABP1; a protein required for the 3′end formation of mRNAs. Interestingly, hCLE also interacts with Nup153, which is essential for the nuclear pore localization of Nup50 and whose interactions with soluble transport factors underlie the efficiency of certain nucleocytoplasmic transport pathways [36]. On the other hand, cytoskeletal-related proteins and translation-related proteins that belong to the 40S and 60S ribosomal subunits are also found associated to hCLE both in the nucleus and the cytosol (Table 3). Remarkable is the presence of the nuclear fragile X mental retardation-interacting protein 2 (FIP-82), a novel FMRP-interacting protein [37], in the cytosolic-hCLE interacting proteins, FMRP is a component of mRNP complexes found in association with actively translating polyribosomes, RNA complexes trafficking in neurites, and RNA granules in the cytoplasm [38].

The association of hCLE with specific proteins in both nuclear and cytoplasmic compartments deserves a special mention. One of these proteins is a motor-related protein, the myosin light chain kinase II. It phosphorylates myosin II and modulates its activity, a motor protein and a driving force behind active transport in the cytoplasm [39]. Additionally, its nuclear and cytosolic-hCLE association is in agreement with a role in the nucleo-cytoplasmic traffic of the hCLE-RNA-containing complexes. Other nuclear and cytosolic hCLE-interacting proteins are DDX1, HSPC117 and FAM98. DDX1 is an RNA-helicase that binds homopolymeric poly(A) RNA [40] and modulates HIV-1 replication [41]. Very little information is available about FAM98B and HSPC117 proteins, both are found associated together with hCLE in nuclear tRNA ligase complex [6], [42], and in cytoplasmic kinesin-associated RNA-transporting granules in dendrites [8], supporting the linkage of nuclear and cytoplasmic phases in RNP metabolism although the functional connection between their nuclear and cytosolic protein complexes remains elusive.

### HCLE is a Shuttling Protein whose Import Depends on Active Transcription

The hCLE-PAGFP fusion protein has permitted us to carry out live cell fluorescence microscopy that demonstrates that hCLE is a shuttling protein. At the molecular level there is no evidence of nuclear localization signals (NLSs) in the hCLE sequence, although putative nuclear export signals (NESs) have been found. Some shuttling proteins accumulate in the cytoplasm when RNA polymerase II transcription is inhibited [43]. This effect relies on the fact that nuclear reimport requires ongoing transcription, for reasons that are not fully understood. This effect was observed in hCLE when cells were treated with the transcription inhibitor Actinomycin D (Fig. 8–11), but also in HSPC117 and DDX1 (Figs. 10–11). Among the shuttling proteins that require active transcription for their reimport, some are RNA binding proteins that depend on their RNA binding domains for shuttling and belong to protein families like the SR proteins (ASF/SF2) or the hnRNPs (hnRNP A1) [44], [45]. Although hCLE has been described as an RNA-binding protein no recognized RNA binding motifs have been found in its sequence [26]. Other example of protein whose nuclear import is prevented by RNA polymerase II inhibitors is the testis-abundant DAZAP1 RNA-binding protein that has been involved in mRNA transport [46]. The reason why these proteins require on going transcription for their nuclear import has not been examined although it has been suggested that these proteins connect the nuclear and cytoplasmic metabolism of RNA and therefore their nuclear import is only required when new RNA cargo are synthesized. By contrast, the nuclear export of some RNA-binding proteins is inhibited when RNA polymerase II activity is inhibited. Interestingly, PABP1 and the TATA-binding protein-associated factor 2N (TAF15) that are found associated with hCLE in the nucleus or the cytosol respectively, remain in the nucleus when the cells are treated with RNA polymerase II activity inhibitors [47], [48]. TAF15 was initially identified as an RNA-binding protein and a TFIID and RNA polymerase II associated factor [49], while PABP1 is a multifunctional protein that participates in the 3′ end formation of the mRNA. The described requirement of ongoing transcription for the nuclear exit of these proteins has been ascribed to their participation in nuclear events associated with the formation and transport of mRNP to the cytoplasm.

Collectively, we have shown that endogenous hCLE shares some features with DDX1, HSPC117 and FAM98B, proteins that associate with nuclear and cytoplasmic purified hCLE. Among them, the four proteins fractionate in protein complexes of similar size in the nucleus and the cytoplasm, together with the S6 ribosomal protein, being the size of the corresponding complexes depending on the presence of RNA. Moreover, nuclear accumulation of DDX1, HSPC117 and FAM98B requires ongoing transcription, as is the case for hCLE and in addition DDX1, HSPC117 and FAM98B expression is modulated by hCLE since its silencing provokes their nuclear and cytosolic down-regulation. These data together with the presence of ribosomal and cytoskeleton-related proteins associated to purified hCLE, support a model in which the cytosolic hCLE-DDX1-HSPC117-FAM98B complex would enter the nucleus in conditions of active transcription then it would associate to newly synthesized RNAs mediating their nucleo-cytoplasmic transport and subsequently may modulate the translation of the associated RNAs. Extensive studies about the generation of this complex and its nuclear and cytosolic remodeling by additional associated proteins are required to assess the proposed key role of the core complex in the modulation of RNA synthesis and fate.

## Supporting Information

Video S1 **Cultured HEK293T cells were transfected with the phCLE-PAGFP (photoactivatable GFP) plasmid and 24 h post-transfection they were used for live cell microscopy.** The video was analyzed with Leica Microsystems LAS AF software (version 2.3.0). Photoactivation was applied in the cytosol to visualize hCLE import.
(AVI)

Video S2 **Cultured HEK293T cells were transfected with the phCLE-PAGFP (photoactivatable GFP) plasmid and 24 h post-transfection they were used for live cell microscopy.** The video was analyzed with Leica Microsystems LAS AF software (version 2.3.0). Photoactivation was applied in the nucleus to visualize hCLE export.
(AVI)

Video S3 **Cultured HEK293T cells were transfected with the phCLE-PAGFP (photoactivatable GFP) plasmid and 24 h post-transfection they were treated with Actinomycin D during a 10 min pulse, washed and used for live cell microscopy**. Photoactivation was applied in the cytosol to visualize hCLE import.
(AVI)

Video S4 **Cultured HEK293T cells were transfected with the phCLE-PAGFP (photoactivatable GFP) plasmid and 24 h post-transfection they were treated with Actinomycin D during a 10 min pulse, washed and used for live cell microscopy.** Photoactivation was applied in the nucleus to visualize hCLE export.
(AVI)
